# Models of osteoarthritis: the good, the bad and the promising

**DOI:** 10.1016/j.joca.2018.09.016

**Published:** 2019-02

**Authors:** P.J. Cope, K. Ourradi, Y. Li, M. Sharif

**Affiliations:** Musculoskeletal Research Unit, Translational Health Sciences, Bristol Medical School, University of Bristol, Learning and Research Building Level 2, Southmead Hospital, Bristol, BS10 5NB, UK

**Keywords:** Osteochondral plugs, Osteoarthritis, *ex-vivo* model, *in-vivo* model, Animal-model

## Abstract

Osteoarthritis (OA) is a chronic degenerative disease of diarthrodial joints most commonly affecting people over the age of forty. The causes of OA are still unknown and there is much debate in the literature as to the exact sequence of events that trigger the onset of the heterogeneous disease we recognise as OA.

There is currently no consensus model for OA that naturally reflects human disease. Existing *ex-vivo* models do not incorporate the important inter-tissue communication between joint components required for disease progression and differences in size, anatomy, histology and biomechanics between different animal models makes translation to the human model very difficult. This narrative review highlights the advantages and disadvantages of the current models used to study OA. It discusses the challenges of producing a more reliable OA-model and proposes a direction for the development of a consensus model that reflects the natural environment of human OA.

We suggest that a human osteochondral plug-based model may overcome many of the fundamental limitations associated with animal and *in-vitro* models based on isolated cells. Such a model will also provide a platform for the development and testing of targeted treatment and validation of novel OA markers directly on human tissues.

## Introduction

Osteoarthritis (OA) is a chronic degenerative disease of diarthrodial joints, predominantly affecting the spine and peripheral joints of the body, particularly the hands, hips, knees and feet. OA most commonly affects people over the age of forty, with the risk of disease increasing with age. OA is a complex heterogeneous disease with different clinical and biochemical phenotypes.

The cause(s) of OA are unknown, and many studies have suggested that the pathobiology of OA is far more complex than a simple cartilaginous or bone disease. It is now acknowledged that OA affects many joint structures, including degeneration of cartilage, abnormal bone remodelling and synovial inflammation^1^, ^2^. Also, studies have shown that there is a complex interplay between the different joint components, making understanding of the degradative sequence of events involved in OA pathogenesis very difficult to dissect^3^, ^4^, ^5^.

The initial onset of OA disease is considered due to an imbalance between the cartilage degradation and repair process^6^, ^7^. The exact sequence of events that trigger the onset of the disease is however widely debated throughout the literature. One hypothesis, suggests that secretion of pro-inflammatory cytokines into the synovial joint induces matrix metalloproteinases which cause the fragmentation and degradation of cartilage extracellular matrix leading to bone remodelling and synovitis^8^, ^9^, ^10^. Contrary to this theory, some studies suggest that subchondral bone remodelling and synovitis precede articular degeneration in the early stages of OA^8^, ^11^, ^12^. While other studies suggest that meniscal degeneration evolving through fibrillation of tissue and a decrease in the levels of type I and II collagen within the meniscus, act as a predisposing or contributing factor to OA progression^13^, ^14^. In the later stages of OA, formation of subchondral cysts, subchondral sclerosis and osteophytes occur as a direct result of bone remodelling, cartilage degradation and synovitis^15^, ^16^, ^17^.

Treatment of OA is largely symptomatic due to insufficient understanding of aetiopathogenesis hindering the development of suitable disease-modifying drugs. This makes targeted treatment of OA a distinct challenge. Human OA tissue samples are usually collected for research once end stages of the disease have been reached, for example during joint replacement, by which time destructive changes in the joint are well established. This makes studying the early disease process very challenging^18^. OA pathology, particularly early OA, is therefore very difficult to study, and so researchers turn to *in-vivo* and *ex-vivo* preclinical animal models to investigate early pathological changes in OA. These models offer unique advantages as well as limitations for studying human OA. This article will review the different models used for investigation of OA, discuss their advantages and disadvantages, and propose development of a gold standard model for OA that closely reflects natural human disease.

## Current models used in OA research

OA research models can be categorised into either *ex-vivo* or *in-vivo* models. Depending on the research question, different models can be used to address different aspects of OA development and progression. Each model has its advantages, yet it has become clear that no single model provides the opportunity to study the disease as a whole. The different models currently used in OA research are discussed below.

### Ex-vivo models

*Ex-vivo* models can be categorised into monolayer culture, co-culture, three-dimensional (3D) culture and explant-based culture. Each model has its advantages and disadvantages and so can be used to answer different questions in OA research.

*Ex-vivo* models such as monolayer culture and co-culture are easier and cheaper to produce than 3D cell cultures and explant-based models. Monolayer cultures are also easy to produce on a large scale and avoid the challenges associated with culturing different cell types at different conditions. However, monolayer and co-cultures are limited in their use due to the fact that they isolate only one or two tissue components at a time. Many studies have shown that there is a strong interplaying network of communication between different joint components that help regulate and maintain a healthy joint, and so isolation of specific joint components hinders this communication^3^, ^19^, ^20^. For example, healthy articular cartilage is dependent upon the release of soluble factors by subchondral bone, and interactions between chondrocytes and synovial fluid ensures the flow of growth factors, regulatory peptides and nutrients between them^19^, ^20^. When injured cartilage is co-cultured with synovium, a protective effect is produced on the synoviocytes^21^. Similarly, culture of subchondral bone and cartilage separately results in increased chondrocyte death and cartilage degradation as well as decreased protein content in culture media compared to when cultured together^3^, ^19^, ^22^. Explant models and 3D cell cultures allow for this inter-tissue communication and so are arguably more useful models available to OA researchers to reproduce natural *in-vivo* environments. Despite this, these models are more difficult to produce in terms of tissue volume and maintaining cell viability over extended periods of time. Some of the advantages, disadvantages and applications of various *ex-vivo* models used in OA research are summarised in Table I.

**Table I:** A summary of the advantages and disadvantages of different *ex-vivo* models used in OA research

*Ex-vivo* Model	Advantages	Disadvantages	Example of the application of the model in OA research
Monolayer culture	- A large number of cells can be easily produced from a single sample^23^ - The configuration of cells cultured in a monolayer layout allows homogenous spread of nutrients and growth factor from the culture medium^24^	- Limited for certain tissue types such as cartilage, whose phenotype changes once in a monolayer culture environment, introducing inter-experimental variability^25^, ^26^ - Chondrocytes are very sensitive to their molecular environment and so need to remain in contact with the extracellular matrix to ensure that they reflect natural *in-vivo* samples^23^ - Cartilage has low cellularity, therefore, a large sample of cartilage is required to ensure sufficient numbers of cells are present to carry out a reliable experiment^23^ - Isolating a tissue in culture removes all systemic influences on that tissue, which does not reflect natural joint tissue - Cells in monoculture traditionally grow on a flat surface in glass or plastic flasks and so do not allow for growth in all directions, as seen in the natural 3D *in-vivo* environment^24^	- Monolayer cultures can be used to study the effects of cytokine stimulation and osmotic pressure^23^ - Synovial cell cultures useful to study the role of the synovium in OA
Co-culturing cells	- Co-culturing cells of different lineages is important to allow for changes in cell-specific physiology and cell–cell interactions that are important in regulating cell and tissue physiology^23^, ^27^	- Different conditions are required for culturing each cell type^23^ - Co-culturing cells can result in alterations of phenotype when cells are isolated^23^ - Co-cultures traditionally grow on a flat surface in glass or plastic flasks and so do not allow for growth in all directions, as seen in the natural 3D *in-vivo* environment^24^	- Co-culturing cells can be used to study the effects of cytokine stimulation and osmotic pressure^23^ - Osteoblast-chondrocyte co-culture useful in understanding bone-cartilage cross-talk^28^ - Co-culturing chondrocytes and osteoblasts results in greater cell growth, matrix production and deposition as well as reduced glycosaminoglycan deposition compared to culturing chondrocytes alone^29^, ^30^ - Co-culturing sclerotic osteoarthritic osteoblasts and chondrocytes from osteoarthritic articular cartilage results in an increased shift towards chondrocyte hypertrophy and release of matrix metalloproteinases and aggrecanases^31^, ^32^ - Culturing synovium and cartilage together produce very different results in terms of the break-down of proteoglycan and matrix structure compared to when cultured alone^4^ - Co-culturing synovium and injured cartilage produces a protective effect on synoviocytes^21^ - Synovium-cartilage cultures useful to study the role of the synovium in OA - Co-culture of bone components ensure balanced bone remodelling^5^
3D cell culture	- 3D cell culture allows for culture of different cell lines and important cell–cell interactions - 3D cell cultures grow as aggregates or spheroids in a matrix, allowing growth in all directions, similar to the natural *in-vivo* environment^24^ - The 3D structure provides structural strength to sensitive cells^23^	- The proliferation rate of cells tends to be slower in 3D cell cultures compared to 2D cultures^33^ - The structural strength provided to cultured cells depends on the scaffold used^23^	- 3D cell culture can be used to study the effects of cytokine stimulation and osmotic pressure, as well as the effects of physical injury and loading on tissue^23^ - A matrix structure of collagens and proteoglycans favours phenotypically normal cartilage^28^
Explant based models	- Simple, cheap and easy to produce^23^ - Explant models allow for the natural processes that occur within the extracellular matrix environment to be observed^26^	- Cell death often occurs at the explant edge - Only a limited number of cells can be extracted from a single source - Limited tissue availability and significant inter-experimental variability^23^	- Explant based models can be used to study the effects of cytokine stimulation and osmotic pressure, as well as the effects of physical injury and biomechanical loading on tissue^23^, ^28^ - Synovial tissue explants useful to study the role of the synovium in OA

### *In-vivo* models

Many animal models in at least eighteen different species have been developed to study established pathological features of OA such as pain, synovitis, cartilage degeneration and bone remodelling. Animal models used in OA research (see Table II) can be categorised into either induced or spontaneous models. Induced models refer to models where OA disease (or OA like features) have been induced either chemically or surgically. On the other hand, spontaneous models are subcategorised into naturally occurring and genetically modified models that develop OA.

**Table II:** A summary of the different animal models used in OA research

Species/Model	Spontaneous	Surgically induced	Chemically Induced	Examples of the application of the model in OA research
Mouse	Naturally occurring OA^1^, ^2^, ^34^ - Genetic models: PAR2^−/−^, CD4^−/−^, MMP17^−/−^, Tenascin C^−/-^, Ddr2^−/−^, SulPhatase^−/-^ 1/2, Syndecan 4^−/−^, Fgf2^−/−^, Mmp13^−/−^, Hif2a^+/-^, GDF5^+/−^, Osteopontin, Ptges1, Tnfrsf11b^+/-^, Runx 2^+/−^, ADAMTS-5/4 ^−/−^, Adamts5^−/−^, ADAMTS4^−/−^, MMP3^−/−^, ICE^−/-^, IL-1β^−/-^, iNOS ^−/−^^35^ - Transgenic models^2^, ^36^ Mutations in type II collagen gene^1^ Brtl mouse^37^ Mouse Del1: Short deletion in type II collagen^18^ Col9a1 knockout^36^ STR/ORT + C57/BL6 strains^1^, ^2^, ^36^, ^38^, ^39^, ^40^	- Anterior cruciate ligament transection (ACLT)^18^, ^34^, ^41^, ^42^, ^43^ - Articular groove model^34^ - Intra-articular tibial plateau fracture, cyclic articular cartilage tibial compression, anterior cruciate ligament, rupture via tibial compression overload^2^ - Ovariectomy^34^ - Partial discectomy^44^ - Medial partial meniscectomy^34^ - Destabilisation of medial meniscus, meniscectomy, tibial overload, fracture models^34^ - Meniscal destabilisation^34^, ^38^, ^40^, ^43^ - ACLT and removal of medial/lateral meniscus or transection of posterior/medial/lateral collateral ligament^34^	- Mono-iodoacetate (MIA) intra-articular injection^34^, ^40^ - Steroids, cytokines^34^, ^40^ - Papain^34^, ^40^ - Collagenase^34^, ^40^, ^41^	- Mouse models widely used for toxicology testing.^1^ - Mouse models used to study the molecular basis of OA.^45^ - Genetically modified mouse models used to investigate the genetic factors and specific genes involved in cartilage degeneration, bone remodelling and inflammation^2^, ^18^, ^43^, ^45^
Rat	- Naturally occurring OA uncommon^18^, ^41^	- ACLT^34^, ^41^, ^46^ - Medial meniscectomy (MMx)^34^, ^40^, ^41^, ^46^ - Articular groove model^34^ - Medial meniscal transection (MMT)^1^, ^34^, ^41^, ^45^ - Combination surgery^40^ - Ovariectomy^34^, ^40^, ^41^ - Partial medial meniscectomy^45^ - Immobilization^40^ - ACL injury^47^	- Intra-articular injection of steroids, cytokines^40^ - Collagenase^40^, ^41^ - Iodoacetate injection^18^, ^34^, ^40^, ^41^, ^45^, ^48^ - Papain^34^, ^40^, ^41^ - Immunotoxin^41^	- Rat model useful in toxicology testing of pharmaceutical compounds^1^. MMT, medial collateral ligament transection (MCLT) and iodoacetate induced models used to study pain^40^, ^45^ - Rat undergone partial medial meniscectomy are useful in cartilage restoration techniques^45^
Syrian hamster	- Naturally occurring OA^1^, ^34^ - Transgenic models^1^			- Syrian hamster OA models are naturally occurring, and transgenic models are used to study pathogenesis of OA^1^
Guinea pig	- Naturally occurring OA^2^, ^34^, ^39^, ^40^, ^41^, ^45^, ^49^ - Transgenic models^1^ - Naturally occurring OA in medial compartment of knee joint in Dunkin Hartley guinea pigs^1^, ^18^	- ACLT^40^, ^41^ - MCLT, osteotomy, patellectomy, sciatic neurectomy^41^ - Meniscal transection^1^, ^34^ - Ovariectomy^34^, ^41^ - MMx^41^, ^50^ - Combination surgery^40^	- Immunotoxin, papain, collagenase, copper II bisglycinate, lipopolysaccharide, chondromucoprotein^41^ - MIA^34^, ^41^ - Quinolone^34^	- Transgenic guinea pig models used to study pathogenesis of OA^1^ - Guinea pig models used to study age and BMI associated risk factors in OA^49^. Dunkin Hartley guinea pig used in therapeutic and pathogenic studies of knee OA^51^. Guinea pigs induced by medial meniscal tear and spontaneous OA models used to study slow and rapidly progressive OA^34^
Cat		- ACLT^40^		- Useful in pain studies^2^
Rabbit	- Naturally occurring OA^2^, ^52^	- ACLT, MMx, posterior cruciate ligament transection (PCLT), patellectomy^34^, ^39^, ^40^, ^41^ - ACL tear^45^ - Section of medial collateral and both cruciate ligaments, resection of medial meniscus^53^ - Immobilization^40^ - Combination surgery, impact loading, cartilage scarification^40^ - ACLT, ACLT and PCL/MCL^34^ - Ovariectomy^34^ - Articular groove^34^ - Partial and MMx^1^, ^34^, ^39^, ^45^ - Transarticular mechanical impact on patellofemoral joint, femoral condyle impact^2^	- Intra-articular injection of steroids and cytokines^40^ - Papain^34^, ^40^, ^54^, ^55^ - Allogeneic cartilage particles^56^ - Iodoacetate and collagenase^40^, ^41^ - Quinolone^34^ - Chymopapain, trypsin, IL-1β, chondroitinase, vitamin A, fibronectin fragments^41^	- Rabbit models useful in efficacy testing of various compounds such as hyaluronic acid^45^. - Partial meniscectomy models used in testing chondroprotective agents^1^
Canine	- Naturally occurring OA^2^, ^34^, ^41^	- Abrasion, valgus osteotomy, pelvic osteotomy, cartilage defect^41^ - Cranial cruciate ligament transection^57^ - Articular groove^34^, ^41^, ^58^ - ACLT^1^, ^2^, ^41^, ^45^, ^58^, ^59^ - Partial medial and MMx^34^, ^41^ - Immobilization^1^, ^40^ - Impact loading, cartilage scarification^40^ - ACLT^60^ - Groove model in femoral condyle - Transarticular impact to stifle^34^, ^58^	- MIA, papain, calcium pyrophosphate crystals^34^, ^41^ - Allogeneic cartilage particles^61^	- MMx model useful in toxicology testing and ACLT model used to study slow progression of OA and pathogenesis that mimics naturally occurring disease^1^ - Canine models that naturally develop OA have been used in therapeutic intervention preclinical trials^2^ - Transarticular impact models were used to identify whether osteoarthritic changes originate from cartilage or subchondral bone changes^34^ and to study early changes in OA in articular cartilage due to joint impact trauma^34^ - ACLT induced model have been used in identification of OA biomarkers^45^
Caprine	-Naturally occurring OA^41^	- MCLT, ACLT^41^ - MMx^34^, ^39^, ^41^, ^45^, ^62^ - Articular groove^34^ - Unilateral medial MMx, unilateral MCL, meniscal transection, cartilage scarification, unilateral ACLT^63^		- Goat models used to study cartilage repair^45^
Ovine	- Naturally occurring OA^2^	- Lateral meniscectomy, ACLT, MCLT^41^ - Articular groove model^34^ - MMx^34^, ^39^, ^40^, ^41^, ^45^, ^62^ - Bilateral and unilateral Mx, unilateral ACLT, medial MMx, unilateral MCLT, unilateral radial meniscal tear, unilateral caudal pole hemi-meniscectomy, unilateral medial meniscectomy^63^ - Ovariectomy^34^, ^40^		- Ovine models used to study early OA cartilage changes, meniscus changes and related treatment techniques^45^, ^64^
Equine	- Naturally occurring OA^2^, ^45^ - Post carpal fracture, exercise-induced^41^ - Trauma to medial femur and tibia^65^	- Metacarpophalangeal ligament transection^41^ - Osteochondral fragment^66^ - Articular groove model^34^	- Amphotericin, *E*. *coli* lipopolysaccharide, IL-1, carrageenan^41^ - Monosodium iodoacetate^40^, ^41^, ^48^ - Filipin^34^, ^41^, ^67^ - Lipopolysaccharide^68^ - Amphotericin^69^ - Polyvinyl alcohol foam particles^41^, ^70^ - Papain^40^ - Intra-articular injection of steroids, collagenase, cytokines^40^	- Equine models used to study articular cartilage repair, osteochondral defects and naturally occurring bone remodelling^2^
Zebrafish	- Genetic knockout e.g. COL10A1^71^			- Zebrafish model useful in studying gene related pathology of OA^71^
Porcine	- Post-fracture^72^	- ACLT, ovariectomy, ACL reconstruction, articular groove model^34^ - Arthroscopy - Cartilage resurfacing Surgically in miniature pigs: - ACLT and ACL reconstruction^41^		- Porcine model used to study repair and regeneration of focal cartilage defects^73^
Bovine	- Naturally occurring OA in patella^74^, ^75^	- ACLT^34^		
Non-human primates	- Naturally occurring OA^1^, ^34^, ^76^ - Naturally occurring OA in macaques^34^, ^40^, ^77^, ^78^ - Transgenic models^1^	- Meniscectomy^34^, ^40^ - Ovariectomy in macaques^40^, ^77^, ^79^ - Ovariectomy in cynomolgus monkeys^34^	- Collagenase induced in cynomolgus monkeys^80^	- Naturally occurring and transgenic models of non-human primates used to study general features of OA^1^

Smaller animal models of OA such as mice, rats, rabbits and guinea pigs are much easier, quicker, cheaper and more readily available than larger animal models such as horses, pigs and dogs^2^, ^40^. Smaller animals can be handled and housed with greater ease than larger models, but due to their smaller size, tissue samples extracted are much smaller and therefore tend to differ to a greater extent in their anatomical and histological structure when compared to humans^18^. Larger animal models therefore provide many advantages over the use of smaller animal models in terms of their greater anatomical similarity to the human model. A dog's articular cartilage for example is half the thickness of a humans, whereas that of a mouse is a minimum of 70 times thinner^2^, ^18^. Additionally, a wider range of tests can be performed on larger animals, such as repeated synovial fluid collection and imaging. They also have a longer life span allowing for slower disease progression and time to end stage OA, as seen in humans. Whilst slow progressive models most accurately reflect human OA, they are however more expensive and time consuming to conduct. There are also greater ethical considerations around the use of larger animal models such as non-human primates and canines^41^. Based on this, some animal models are therefore better suited to OA research than others, such as the canine, caprine, bovine and porcine models. Some of the advantages and disadvantages of various animal species used in OA research are summarised in Table III.

**Table III:** A summary of the advantages and disadvantages of different animal models used in OA research

Animal Model	Advantages	Disadvantages
Mouse	- Mice have a short life span (generally one or 2 years) and so develop OA fairly rapidly, making mice an easy model to study the whole disease process^18^, ^43^ - Small animal size means the whole joint can be histologically sectioned^81^ - Mice are easily managed, with low maintenance cost, demonstrate rapid disease onset and their complete genome is available for study^40^ - Genetically modified mouse models are easy to produce and are useful to investigate the genetic factors involved in OA pathogenesis, specifically genes involved in cartilage degeneration, bone remodelling and inflammation^18^, ^45^ - Mouse models can be used in toxicology testing and to establish the molecular basis of OA^1^, ^45^	- Huge variation in results observed between different strains of mice^18^ - Disease severity varies with age, with older mice more representative of human disease^82^ - Difficult to ascertain skeletal maturity as growth plates often do not close completely^82^ - Mice are anatomically and histologically different to humans, for example, mice have a thicker layer of calcified cartilage, do not have three distinct chondrocyte layers and have a cartilage seventy times thinner than humans^45^ - Macroscopic lesions and degrees of damage are difficult to identify due to the small anatomical size of mice^81^ - The progression and process of disease is faster in mice than in humans (weeks rather than decades)^36^ - The small size of mice makes surgically inducing OA more challenging^40^ - Postoperative management of mice is difficult in surgically induced models^40^, ^45^
Rat	- Rat cartilage is thicker than that of mice, so it is possible to induce partial and full-thickness cartilage defects^1^, ^45^ - Rats rarely experience post-operative infection so are useful animal models to surgically induce OA^1^ - Rats are easily managed and require low maintenance costs^40^, ^45^ - It is easier to perform surgery in rats than in mice due to their larger size^40^ - The full rat genome is available for study^40^ - MMT, MCL transection and iodoacetate models useful to study pain^40^, ^45^ - Rat models useful in toxicology testing and studying cartilage restoration techniques^1^, ^45^	- Naturally occurring OA is uncommon in rats, variation in results is often observed between different strains of rat and disease severity varies with age, with older rats tending to present with more severe OA^18^ - It is difficult to ascertain the skeletal maturity of rats^83^ - Rats have greater volumes of highly vascularised adipose tissue and muscle in the medial knee region - Post-operative rats immediately resume load-bearing which accelerates joint degeneration^1^ - Genetically engineered rat models are not available and postoperative management of rats is challenging^45^
Guinea Pig	- The guinea pig model has similar OA histopathology to disease in humans^84^ - Guinea pigs are large enough that tissue samples can be easily collected for tests and the whole joint can be histologically sectioned^49^ - Guinea pigs are easy to manage^40^ - Naturally occurring guinea pig models are available and the disease pathogenesis is predictable and similar to that seen in humans^1^, ^45^ -Hartley guinea pigs can be used to study risk factors for OA such as BMI and age - Complete guinea pig genome available^85^	- The weight of each guinea pig and whether they are housed alone or in pairs influences the severity of their OA^41^, ^49^ - Unlike in humans, guinea pigs resume load bearing post-operatively which accelerates joint degeneration^1^ - The time to guinea pig skeletal maturity is fast^45^
Cat	- Cats are larger in size allowing for tissue and fluid collection^40^ - The full cat genome is available^40^	- Cats are difficult and costly to manage and there are ethical issues surrounding emotional attachment^40^ - Cats display genetic variability between individuals^40^
Rabbit	- Naturally occurring OA is very common in rabbits^52^ - Rabbit model useful in studying the efficacy of compounds^45^ - Complete rabbit genome available^86^	- Rabbits have a very different gait compared to humans and only rabbits over the age of eight or 9 months can be used to guarantee skeletal maturity^52^ - The cartilage of rabbits is ten times thinner compared to humans, with a higher chondrocyte density and cartilage zonal layers that varies highly within the same joint^87^, ^88^ - The rabbit meniscus is more cellular, has less vascular penetration and can heal faster than the human menisci^89^ - Rabbit cartilage can spontaneously heal and regenerate and there is no complete rabbit genome available for study^40^ - OA progression varies with the age of the rabbit after surgical OA induction, with faster progression seen in older rabbits^41^
Canine	- Canines have similar anatomy and disease progression to humans^18^, ^90^ - Canines display a widespread clinical incidence of OA^18^, ^83^ - Canines are easy to manage and train postoperatively^40^, ^45^ - Surgical lesions develop slowly in canines, similar to the human model^1^ - Canines have similar gastrointestinal physiology to humans^45^ - The canine model is widely used so comparison across different studies can be made, the larger size of canines allows for tissue and fluid collection and the full canine genome is available^40^ - Naturally occurring OA models are available for intervention preclinical trials^2^, ^41^	- Canines have different joint biomechanics and gait compared to humans, their skeletal maturity is not reached until 9 to 18 months of age and their cartilage is half the thickness of human cartilage^64^ - There are ethical issues surrounding emotional attachment of dogs and management is costly^40^, ^45^ - Canines display genetic variability between individuals^40^
Caprine	- Anatomically the caprine stifle joint is very similar to the human knee^64^ - The caprine stifle joint is closest in size to the human knee joint, the larger size of the animal allows for tissue and fluid collection and goat cartilage thickness is close to that of humans^40^ - Goats are cheap and easy to use in studies compared to most large animal models and they can be used to study cartilage repair^45^ - Complete goat genome available^91^	- Caprine cartilage thickness varies between individuals, the skeletal maturity of a goat is not reached until at least 2 years of age and cartilage healing capacity varies with a goat's age, with better capacity in younger animals^87^, ^92^ - Cartilage repair outcomes differ in the short and long term and so follow up is required to assess progress^83^ - Naturally occurring OA in goats is uncommon^40^, ^45^
Ovine	- Sheep are cheap and easy to use in studies compared to most large animal models. - The advantages of the sheep model are similar to the caprine model of OA	- The disadvantages of the sheep model are very similar to the caprine model of OA
Equine	- The large size of horses allows for easy tissue and fluid collection and a full genome is available^40^ - Anatomically and histologically the equine stifle joint is similar to the human knee, the articular cartilage is very similar in thickness and the cellular structure, biochemical makeup and properties of the cartilage are most comparable to humans^2^, ^92^, ^93^ - There are a wide range of imaging and clinical tests that can be performed on horses, including rehabilitation techniques^94^ - Naturally occurring OA models are available^45^	- Horses are difficult and expensive to house and manage due to their large size^40^
Zebrafish	- Zebrafish model is useful to study gene related pathology of OA and zebrafish genome available^71^	- Zebrafish do not have synovial joints^71^
Porcine	- The porcine stifle joint is anatomically similar to the human knee joint and pigs have similar immune systems and gastrointestinal physiology to humans^41^ - Pigs are most similar to humans in terms of their anatomy, neurobiology, cardiovasculature, gastrointestinal tract and genome^73^ - Genetically modified models are available and pigs are a useful model to study repair and regeneration of focal cartilage defects^73^ - Pigs have similar joint size, weight-bearing and cartilage thickness to humans^73^	- The porcine meniscus is wider, and the cruciate ligaments are longer than in humans^64^, ^95^ - Pig skeletal maturity is reached between 10 and 24 months of age^41^
Bovine	- Bovine cartilage thickness, cellularity and zonal cartilage layers of patella is similar to in human femoral condyles^75^, ^88^ - Bovine meniscus is biomechanically similar to the human meniscus^95^ - Complete bovine genome available^96^	- Bovine lateral tibial plateau cartilage is thinner, more cellular and varies in zonal cartilage thickness compared to the human^75^, ^88^
Non-human primates	- Non-human primates have similar anatomy, genetics, biology, behaviour and physiology to humans^2^ - The pathology of OA and the relationship between age and disease severity is very similar to in humans^18^, ^76^, ^77^ - The larger size of non-human primates allows for tissue and fluid collection and the full primate genome is available for some species^40^	- Non-human primates are expensive and ethically difficult to keep, for example chimpanzees display depression and post-traumatic stress disorder on a similar scale to that of humans^97^ - Non-human primates have a long-life span and so a long disease pathogenesis time scale which is both time consuming and costly^2^ - There are difficulties in obtaining adequate subject numbers for studies^1^ - Housing and management of non-human primates is challenging^40^

#### Challenges presented by current OA models

At present, there is no gold standard animal model used in OA research. Differences in size, anatomy, histology (specifically cartilage thickness) biomechanics and physiology makes translatability between animal models and human disease very difficult^52^, ^83^. Challenges are posed by species-specific differences in disease pathology and progression, as well as normal joint homeostasis, specifically the repair processes that occur within the joint. Different OA induction methods used in certain animal species also sometimes results in differences in OA presentation. There is therefore a need to reduce experimental variability and increase the reliability of data interpretation. Furthermore, different animal models have been shown to represent different stages of the disease more effectively, making selection of an animal model that completely reflects natural human disease challenging. To add to this, it has become clear that there is much dispute as to what defines OA and which molecules are associated with the disease. This is in part hindered by the fact that current *ex-vivo* models do not allow for the important inter-tissue communication between different joint components required for natural OA disease processes to be studied. To gain a better understanding of the mechanisms of joint damage in OA, specifically the exact sequence of events and interactions between different joint components, it has been suggested that more focus should be placed on developing 3D cell cultures and explant-based models that allow for these important interactions, providing interesting opportunities for researchers to develop their understanding of OA.

In the ideal animal model, the disease must be induced reliably, with 100% penetrance, and within a suitable time frame, and yet still present with disease characteristics that are comparable to the human condition. Disease progression in the animal model should also allow for the examination of all stages of disease to ensure full detection of any therapeutic effects. The animal must be inexpensive, easy to house and manage but also be of large enough size to allow for a full range of analysis techniques to be performed. The animal must also be anatomically, biomechanically and histologically similar to humans^40^. Some animal species, such as the canine, caprine, bovine and porcine models, are therefore better suited to OA research than others.

#### A promising model for OA

OA is a disease of the whole joint and therefore the gold standard model for human OA must allow for key communication between different tissues of the joint. An osteochondral (cartilage on bone) model may overcome many of the current challenges and limitations of the various models discussed above. The use of osteochondral plugs provides a model incorporating the key joint tissues affected in OA, maintaining the important interactions between these tissues as seen in human disease. There are a few studies that have used osteochondral plugs from bovine, equine and human samples as *ex-vivo* models of OA^98^, ^99^, ^100^. These osteochondral plug-based models appear very promising and so further studies should be encouraged as the basis for developing a gold standard model for OA. In the model design, cytokines such as interleukin-1 beta (IL-1β) and tumour necrosis factor alpha (TNF-α) may be used as the best method for inducing OA (cartilage damage) and tissue responses that closely replicate the natural disease, as these cytokines are known to contribute to the inflammatory effect of the synovium in the model. However, OA is a complex disease and many cytokines and chemokines have been shown to be expressed in OA synovium and detected in synovial fluid. Therefore, in the model design, investigators should consider using synovial tissue and/or synovial fluid from patients with active disease to induce OA in the osteochondral plugs. Osteochondral plugs can be harvested from joint surfaces, such as the femoral condyle, tibial plateau and patella. Methods of plug extraction available for use include different sizes of graft harvester, biopsy punch, mosaicplasty osteotome, diamond tipped cylindrical cutter or surgical trephine burr. Plugs can be cultured in serum-free culture medium such as Dulbecco's Modified Eagle Medium F-12 (DMEMF-12, Invitrogen, USA) or α-Minimum Essential Medium (α-MEM, 22,561 Gibco, The Netherlands) for up to 57 days with significant cell viability^98^. Indeed, an early study reported that >99% chondrocyte viability can be maintained in the untraumatized areas at the centre of the osteochondral plugs^100^.

The availability of an osteochondral plug-based model, particularly a human tissue-based one, would be invaluable in screening of new disease-modifying osteoarthritis drugs (DMOADs). Currently, there are many DMOADs under different stages of development. Early studies of these drugs were in animal models, but the availability of this new model will provide an opportunity to directly test these drugs on human tissues. An osteochondral plug system like this may also be used for discovery of novel markers for OA.

## Conclusion

It is clear that we are limited in our understanding of OA because we do not have a suitable model that accurately reflects natural human OA. Whilst animal models provide crucial information about disease mechanisms, none of the current models used in OA research recreate the natural *in-vivo* environment and allow the whole disease process to be studied. Differences in anatomy and biomechanics also makes translatability to the human model a distinct challenge. It is also important to consider the cost and ease of management of using animal models in research. Whilst smaller animal models provide many benefits in terms of availability, handling and management, larger animal models such as canines and pigs are not only more comparable to humans physiologically but also in their progression to disease. Their larger size also allows for performance of a broader range of analysis techniques and investigations.

The models currently used in OA research each have their advantages and disadvantages; however, it has become clear that there are consistent problems with all of these models that hinders our ability to understand the pathogenesis of OA. The only way to achieve greater understanding of the pathological processes that underpin OA is to produce a ‘gold standard’ model for OA. Development of a consensus model will provide greater understanding of the specific stages and interactions involved in OA pathogenesis, as well as a model that can be used to compare data findings between different research groups, test pre-clinical drugs and identify and test possible biomarker targets directly on OA joint tissue. An osteochondral plug-based model could be a “promising” new model for OA, able to provide a reliable throughput model for proof of concept and mechanistic studies, aiding the discovery of targeted OA therapy. The model would also provide an opportunity to reduce the financial, ethical and time restraints associated with using animals in research, shifting OA research to embody the principle of the 3Rs; replacement, reduction and refinement of animal use in research.

## Author's contributions

Conception and design: MS, KO, YL.

Drafting of the article: PC, MS, KO.

Provision of study materials: PC, YL.

Final approval of the article: MS.

## Competing interest statement

The authors declare that they have no competing interests.

## Funding

Not applicable.
